# Editorial: Muscle oxygenation and vascular adaptations in sports performance and rehabilitation

**DOI:** 10.3389/fphys.2024.1502939

**Published:** 2024-10-14

**Authors:** Rodrigo Yáñez-Sepúlveda, Daniel Rojas Valverde, Jose A. Parraca, François Billaut, Stéphane Perrey, Aldo A. Vasquez-Bonilla

**Affiliations:** ^1^ Faculty Education and Social Sciences, Universidad Andres Bello, Santiago, Chile; ^2^ Núcleo de Estudios en Alto Rendimiento y Salud (CIDISAD-NARS), Escuela Ciencias del Movimiento Humano y Calidad de Vida (CIEMHCAVI), Universidad Nacional, Heredia, Costa Rica; ^3^ Department of Sport and Health, Universidade de Évora, Evora, Portugal; ^4^ Département de Kinésiologie, Faculté de Médecine, Université Laval Québec, Quebec, QC, Canada; ^5^ EuroMov Digital Health in Motion, University Montpellier, IMT Mines Ales, Montpellier, France; ^6^ Facultad de Ciencias del Deporte, Universidad de Extremadura, Cáceres, Spain

**Keywords:** performance, near-infrared spectroscopy, sport injury, vascular adaptations to training, muscle oxygen consumption, health, thermal response

Technological advancements in sports and health have enabled the indirect (and non-invasive) measurement of metabolism, blood flow, and oxygenation at the muscle level, during exercise. This has significantly enhanced our understanding of clinical physiology, as applied to physical activity. The use of near-infrared spectroscopy (NIRS, Perrey et al., 2024) to assess muscle oxygenation, alongside thermography to measure skin surface temperature (Tsk, Sillero-Quintana et al., 2021), has shown potential as tools for identifying physiological adaptations relevant to sport performance and health. However, scientific gaps remain regarding the use of NIRS sensor and Tsk data to ascertain metabolic and vascular enhancements derived from physical exercise. Furthermore, a significant milestone in sports science is the application of vascular methods in the functional rehabilitation of pathological conditions and injury sports. This editorial presents four studies using NIRS and Tsk in performance, health, and physical rehabilitation contexts.

The first study, Arnold et al., examined the pattern and reliability of post-exercise reoxygenation using NIRS (Moxy Monitor, Fortiori Design LLC., Hutchinson, MN, United States). This was performed during an incremental cycling test across four muscle sites: the locomotor muscles vastus lateralis (VL) and rectus femoris (RF), as well as the accessory muscles lumbar paraspinal (PS) and lateral deltoid (DL). The results showed slower reoxygenation kinetics in response to increased workload in the VL, RF, and PS, but not in the DL. As expected, the VL, the primary muscle involved in cycling, demonstrated faster reoxygenation (Shibuya and Tanaka, 2003) and exhibited the greatest reliability than the accessory muscles. These findings show the utility of NIRS in the VL from a reoxygenation kinetics perspective (Maliszewski et al., 2024), suggesting that physiologists can indirectly assess the muscle oxidative capacity to recover from exercise and detect subtle adaptations in athletes (Billaut and Buchheit, 2013).

The second study, conducted by Tandirerung et al., used NIRS (Portamon, Artinis Medical System, Netherlands) with a short exercise and arterial occlusion protocol typically employed to measure mitochondrial capacity in endurance athletes and cardiovascular diseases indirectly (Jones et al., 2017). The novelty of this study lies in its focus on reproducibility in non-athletic adults aged 18 to 60, as skeletal muscle function declines with age and across various disease phenotypes, potentially leading to reduced physical performance, frailty, and loss of independence (Gomes et al., 2017). The results demonstrated good reliability for a “short-rapid” protocol, which involved performing rapid dynamic plantar flexions against a resistance band as many times as possible within 10 s. Following this exercise, short transient arterial occlusions were applied, lasting 5–8 s over a 3-min period (5 s in the first minute and 8 s in the second and third minutes) to track muscle oxygen consumption recovery and estimate oxidative capacity from the recovery time constant (τ) (Southern et al., 2014). The method proposed in this study offers insights into tracking pathophysiological alterations in skeletal muscle function, which is necessary for understanding disease mechanisms, progression, and response to intervention (Coen et al., 2019). The findings are related to impaired blood flow transport capacity, reflected in NIRS values.

The third study, conducted by Rubio-Zarapus et al. explores the use of NIRS to investigate adaptations to two types of training (neuromodulation and high-intensity interval training) in fibromyalgia, which is recognized as a chronic disorder characterized by widespread musculoskeletal pain, premature fatigue, and cognitive impairment (Antunes and Marques, 2022). Muscle oxygen saturation (SmO_2_) at rest appears to be associated with improved strength performance and reduced pain in this population (Villafaina et al., 2023). However, evaluating only resting SmO_2_ without considering the SmO_2_ decrease as a target variable limits the understanding of blood flow redistribution and skeletal muscle metabolism. NIRS can be further applied to assess SmO_2_ dynamics during exercise, as impaired muscle oxygen utilization in individuals with fibromyalgia may affect daily activities such as walking and impact their quality of life (Shang et al., 2012). The study shows that monitoring SmO_2_ decreases with NIRS sensors could serve as a valuable, low-cost, non-invasive method to guide strength training and physical therapy in this population (Melian et al., 2021).

The fourth study, conducted by Trovato et al., assessed thermography-derived knee Tsk to evaluate the effects of static and dynamic warm-ups, as well as a 90-degree change of direction exercise, on the temperature response. Thermal responses are related to blood flow dynamics, with vasodilation leading to an increase and vasoconstriction causing a decrease in Tsk (Brengelmann et al., 1977). The circulatory system’s capacity, through blood vessels, to facilitate blood flow and deliver oxygen and nutrients to tissues and organs, such as skeletal muscles, makes monitoring Tsk valuable for insights in injury rehabilitation processes (Gómez-Carmona et al., 2020; Lamers et al., 2022). Despite some limitations—such as the lack of control over exercise intensity, a recreational sample group, and a generalized warm-up focused on the quadriceps—this study marks a significant step forward in sports science. It demonstrates how thermography can identify changes in the knee tissues based on temperature variation, showing that better physical preparation can be achieved through specific warm-up strategies. Coaches can use this information to customize warm-up protocols that optimize knee temperature to prepare athletes more effectively for subsequent performance and potentially reducing injury risks. Additionally, these findings could be helpful for researchers studying knee thermal responses after various intensity exercises and provide a better recovery tracking system.

In summary, this Research Topic highlights the expanding role and diversification of NIRS and thermography in advancing our understanding of muscle oxygenation and vascular responses in the sport performance and rehabilitation contexts. By providing non-invasive, reliable, and cost-effective methods to assess physiological changes, these technologies offer valuable insights for optimizing performance, guiding rehabilitation, and improving health outcomes in athletic and clinical populations. However, further research is needed to address current limitations and expand the applicability of these tools across diverse populations and training modalities. In the coming years, it is suggested to develop a position stand to unify the criteria for the use of these technologies to address the effects on performance and rehabilitation.
